# Large-Scale Assessment of Function and Disability in Patients with Parkinson’s Disease Using the Functioning Disability Evaluation Scale-Adult Version

**DOI:** 10.3390/ijerph15122788

**Published:** 2018-12-09

**Authors:** Tzu-Herng Hsu, Tsan-Hon Liou, Kuei-Ru Chou, Wen-Chou Chi, Chia-Feng Yen, Hua-Fang Liao, Ing-Jy Tseng

**Affiliations:** 1 Department of Physical Medicine and Rehabilitation, Shuang Ho Hospital, Taipei Medical University, New Taipei City 23561, Taiwan; 17324@s.tmu.edu.tw (T.-H.H.); peter_liou@s.tmu.edu.tw (T.-H.L.); 2 Department of Physical Medicine and Rehabilitation, School of Medicine, College of Medicine, Taipei Medical University, Taipei 11031, Taiwan; 3 Graduate Institute of Injury Prevention and Control, College of Public Health, Taipei Medical University, Taipei 11031, Taiwan; 4 School of Nursing, College of Nursing, Taipei Medical University, Taipei 11031, Taiwan; kueiru@tmu.edu.tw; 5 Department of Occupational Therapy, Chung Shan Medical University, Taichung City 40201, Taiwan; y6312002@gmail.com; 6 Department of Public Health, Tzu Chi University, Hualien City 97004, Taiwan; mapleyeng@gmail.com; 7 Taiwan Association of Child Development and Early Intervention, Hualien City 97064, Taiwan; hfliao@ntu.edu.tw; 8 School and Graduate Institute of Physical Therapy, College of Medicine, National Taiwan University, Taipei 10055, Taiwan; 9 School of Gerontology Health Management, College of Nursing, Taipei Medical University, Taipei 11031, Taiwan

**Keywords:** Parkinson’s disease (PD), World Health Organization Disability Assessment Schedule 2.0 (WHODAS 2.0), Functioning Disability Evaluation Scale-Adult Version (FUNDES-Adult), International Classification of Functioning, Disability and Health (ICF)

## Abstract

This study assesses the functioning and disability related to Parkinson’s disease using the Functioning Disability Evaluation Scale-Adult Version (FUNDES-Adult), based on the World Health Organization Disability Assessment Schedule 2.0 (WHODAS 2.0) in a large-scale database; this study describes, discusses and clarifies the predictive factor of not being in an ambulatory status. Of 7455 patients included in this study, 3561 were not ambulatory and 3894 were ambulatory or assisted ambulatory. Patients with poor walking status revealed higher FUNDES-Adult scores in all domains. Age, modified Hoehn–Yahr stage, living in an institution and the standardized score of FUNDES-Adult domains 1 and 2 were positive independent predictors of the not ambulatory status. The FUNDES-Adult could evaluate multifaceted disability and predict the walking status in patients with Parkinson’s disease.

## 1. Introduction

Even with optimal medical management, Parkinson’s disease (PD) remains an extremely debilitating disease and is a major concern, owing to its global impact. Over 4 million people worldwide are estimated to be diagnosed as having PD; this number is projected to double within the next 20 years [1]. In Taiwan, the average age-standardized prevalence of PD per 100,000 individuals was 147.7 in 2011, with an annual increase of 7.9% [2].

A neurodegenerative disorder affecting middle-aged and elderly people, PD is characterized by dopaminergic and nondopaminergic deficiencies [3,4], causing various nonmotor symptoms, such as sensory (pain and tingling), hyposmia voice, sleep disturbance, depression and anxiety, abnormal executive and working memory-related functions [5], and motor symptoms (e.g., tremors, rigidity, bradykinesia, and disturbance of postural and gait control) [6]. Despite the variety of available surgical and pharmacological treatment options, more than half of the adults with PD living in a community dwelling experience gait disturbances associated with a poor quality of life, increased disease severity and disability, especially in the later PD stages [7].

A disability is a functional limitation arising from the interaction between a person and their social, physical and attitudinal contexts, and is characteristically multidimensional; it is also an individual health problem and a condition that occurs in a particular environment [8]. Patients with PD are limited in terms of their employment, interpersonal communication and social activity; moreover, their leisure activities, daily lives, self-care and self-management capabilities are affected. At present, PD has no cure. Moreover, as the proportion of older people increases globally, the healthcare cost of patients with PD is projected to increase. Thus, PD treatment primarily aims at preserving life expectancy and limiting motor impairments [9,10]. According to an investigation in 28 European countries, PD was the fourth most costly disease among the 12 most prevalent neurological disorders; the costs increase as the disease progresses [11]. Studies have compared the total mean costs based on different modified Hoehn–Yahr (H&Y) stages of PD. The cost in a patient with an H&Y stage of 5 was six times as much than the patient with an H&Y stage of 0, 1 or 2, and was 2.2 and 1.6 times as that of stage 3 and 4, respectively [12,13]. The results signified that poor walking status increases the burden on the patients, society and public health system. Thus, finding out predictive factors of poor walking status in patients with Parkinson’s disease is a matter of cardinal significance. However, barriers to computation or the comparison of all factors exist because of different assessment tools used in past research.

The World Health Organization (WHO) Disability Assessment Schedule 2.0 (WHODAS 2.0), based on the International Classification of Functioning, Disability and Health (ICF), was developed by WHO in 2010. The WHODAS 2.0 is a standardized method for measuring health and disability across cultures [14]. The Functioning Disability Evaluation Scale-Adult Version (FUNDES-Adult) developed by the Taiwan ICF research team was created by modifying the WHODAS 2.0 [15,16,17]. Designed as a generic assessment of the levels of disability experienced in activities, it is directly linked to the ICF and examines disability and health status in the six domains of function: Cognition, mobility, self-care, getting along with others, life activities and participation. In addition, environmental attributes and motor action were evaluated as the FUNDES-Adult [18,19,20].

To date, no large-scale study has used the FUNDES-Adult to evaluate disability levels in patients with PD. The current study analyzed the demographic characteristics of patients with PD, including the levels of their disability in motor action and evaluated multifaceted disability to predict the walking status in patients with PD based on the FUNDES-Adult.

## 2. Materials and Methods

### 2.1. Sample

Preliminary data were obtained from a registry of disability evaluation, functional assessment, and by provision of Taiwanese Social and Family Affairs Administration—a ICF framework–based database established by the Ministry of Health and Welfare in Taiwan [21]. This study was approved by the Joint Institutional Review Board at Taipei Medical University (Approval No. N201805048).

**Figure 1:**
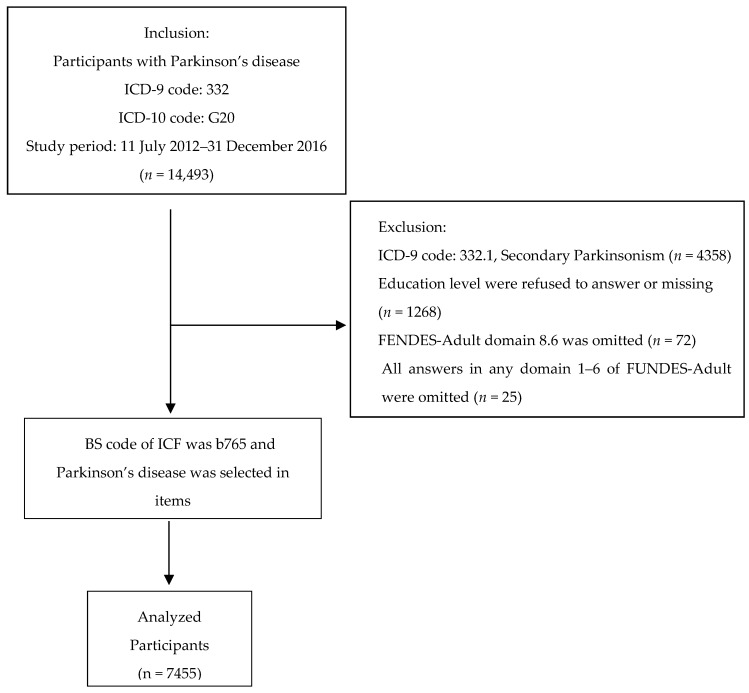
Sample selection flow chart.

Registry applications between July 2012 and December 2016 were collected. Applicants who completed the evaluation procedure and were eventually provided disability benefits by the Government of Taiwan were included. Patients whose data in FUNDES-Adult domain 8.6 were incomplete and FUNDES-Adult domains 1–6 had complete omissions (e.g., six questions in domain 1 were completely omitted) and those who did not receive compulsory education and had no education-related information were excluded. Patients with PD were identified according to the International Classification of Diseases (ICD), Ninth Revision, Clinical Modification (ICD-9-CM) and the ICD, Tenth Revision, Clinical Modification (ICD-10-CM). Data related to PD that were classified elsewhere (ICD-9-CM code: 332, ICD-10-CM code: G20) were selected. The aforementioned patients were included if their body function and structure code of ICF was b765 and PD was selected in items. This yielded 7455 PD cases (3719 men and 3736 women; Figure 1).

### 2.2. Instruments: Domain and Summary Scores of FUNDES-Adult

We administered the 36-item Chinese version of WHODAS 2.0, which was revised as FUNDES-Adult by interviewing the participants (or their proxies if the patients could not respond). The FUNDES-Adult was developed based on the WHO’s ICF, published in 2014 with good validity and reliability [22,23] and used to measure daily life activities and participation. The reliability of the internal consistency and intraclass correlation coefficient values of the FUNDES-Adult are excellent; it ranges from 0.89 to 0.97 (Cronbach’s α, *p* < 0.05) and 0.8 to 0.89, respectively [22,23].

The questionnaire had the following six domains: Cognition (domain 1, six items), mobility (domain 2, five items), self-care (domain 3, four items), getting along with people (domain 4, five items), life activities (domain 5.1, four items for household activities; domain 5.2, four items for school and work activities) and participation in society (domain 6, eight items). In addition, we obtained capability qualifiers data for domain 8.6 (motor action) according to FUNDES-Adult [15,16,21,23]. Respondents rated the extent to which their disabilities interfered with their lives in the preceding 30 days on a 5-point scale ranging from 0 (none) to 4 (extreme/cannot do). Domains 1–6 evaluated the dimension of performance. In domain 8.6 both the dimension of capability and capacity were evaluated and the difficulty level of these two dimensions were judged with or without assistive technology and personal assistance in real life, respectively. For capacity, each patient was requested to walk straight for 3 m and then return to their initial location; for capability, the scores of domains were rated from interviewing.

The domain and summary scores (general disability latent variable) were calculated according to the algorithm of FUNDES-Adult (same as WHODAS 2.0) and converted to 0–100 for each domain and summary score. The lower scores indicated lower difficulty levels. The capacity result for domain 8.6 was substituted by capability qualifiers if the items were missing. Each item would be omitted if the capacity and capability qualifiers were both missing. However, the substitution of the mean (by domain) was used for imputing missing data of the results for domains 1–6. The data were excluded if a question in the domain was not answered.

### 2.3. Procedure

The FUNDES-Adult was evaluated by multiple testers in different hospitals, such as by physical therapists, occupational therapists and social workers, during September 2011 and August 2013 for disability evaluation. They completed on-the-job training and were qualified to conduct the evaluations. The collected data were categorized into four sections: (1) In the first section, we recorded sociodemographic variables such as age, sex, place of residence, work status, education level, family economic status, urbanization level, modified Hoehn–Yahr (H&Y) stage, domain 1 (cognition), domain 2 (mobility), domains 8.6 (walking 3 m and returning) and the final score of each FUNDES-Adult domain. (2) In the second section, we divided data into three groups according to the scores of walking status, comparing differences in sociodemographic variables between each group. (3) In the third section, we applied sociodemographic variables and domain 1 and domain 2 summary scores to walking status through logistic regression. (4) In the fourth section, we finally applied items in domains 1 and 2 to walking status through logistic regression. The scores were determined by trained testers to maximize reliability and consistency. In this article, walking statuses were defined as ambulatory, assisted ambulatory and not ambulatory, according to the FUNDES-Adult scores of domain 8.6 (0, 1–3 and 4, respectively).

Statistical analyses were performed using SAS (version 9.2; SAS Institute Inc., Cary, NC, USA). Sociodemographic data were represented as numbers and percentages (Table 1). Chi-square analysis was used for comparing the categorical variables of sociodemographic data and walking status results. F-tests were used for comparing the standardized FUNDES-Adult scores in the six domains and the raw scores of items in domains 1 and 2 among ambulatory, assisted ambulatory, and not ambulatory groups. To determine risk factors for the not ambulatory status of patients with Parkinson’s disease, standardized FUNDES-Adult scores (shown in Table 3) or FUNDES-Adult Raw Scores (shown in Table 4) and sociodemographic variables (including age, residence, Modified Hoehn–Yahr Stage) were put into binary logistic regression analysis, and not ambulatory status was the result we analyzed. We considered results where *p* < 0.05 statistically significant.

## 3. Results

### 3.1. Sample Characteristics

The main characteristics of the current sample are presented in Table 1. Of the all 7455 patients, 3719 were men and 3736 were women, with most of them being aged age older than 75 years (49.58%; average age of 73.25 years). Most patients lived in community dwelling (87.57%), with 52.45% living in an urban area (35.47% and 12.09% were living suburban and rural areas, respectively). Moreover, 97.65% were unemployed, 53.53% held a primary education level (17.95% were illiterate and 13.90%, 11.40%, and 3.22% had junior high, senior high, and above college-level education, respectively), and 99.01% had an average family economic status. Furthermore, 42.12% and 29.83% were in modified H&Y stages 4 and 3, respectively, and the remaining patients were at stage 5.

The distribution of scores of motor action (walking 3 m and returning) were as follows: “none” (0) 7.98%, “mild” (1) 11.35%, “moderate” (2) 15.13%, “severe” (3) 17.77%, and “extreme” (4) 47.77%. In total, six domains were related to function; after converting the summary score to a metric ranging of 0–100 (where 0 = no disability; 100 = full disability), the average scores from domains 1–6 were 56.76, 80.47, 72.03, 65.68, 87.57, and 57.19, and the summary was 67.17; the mean scores of each item in domains 1 and 2 were 1.50–2.78 and 2.69–3.40, respectively.

Regarding sample representativeness, gender demonstrated no significant difference (*p* > 0.05); however, significant differences were noted for age, residence, work status, education level, family economic status, urbanization level, motor action and modified H&Y stage (*p* < 0.05).

### 3.2. Demographic Data and Walking Status

Demographic and walking status data are listed in Table 2. We divided the participants into three groups: Ambulatory, assisted ambulatory, and not ambulatory groups, consisting of patients whose motor action scores were 0, 1–3, and 4, respectively.

Most patients were not ambulatory (*n* = 3561) and gender showed a significant difference in the ambulatory and not ambulatory groups (*p* < 0.05), but no such significant difference was noted in the assisted ambulatory group. A higher percentage was observed in not ambulatory patients who were aged older than 75 years, resided in an institution, were unemployed, were illiterate and were at a modified H&Y stage 5. In addition, the highest FUNDES-Adult scores in all domains were observed in the not ambulatory group and the results were observed in each question of domains 1 and 2.

### 3.3. Correlation of Demographic Data and Walking Status

In Table 3, we compared the demographic data of the ambulatory and assisted ambulatory groups with those of the not ambulatory group through logistic regression. The analysis revealed that age >75 (OR = 3.342, 95% CI, 1.791–6.235, *p* < 0.0001), residence in institution (OR = 2.735, 95% CI, 2.149–3.48, *p* < 0.0001) and higher stages of modified H&Y were independent factors predicting walking status among patient with PD. In addition, cognition and mobility in the FUNDES-Adult standardized scores revealed a positive correlation with the not ambulatory status.

In Table 4, we excluded the patients whose FUNDES-Adult and domain 1 and 2 items were incomplete. In these sections, similar results, such as age >75 years, residence in institution, unemployment and higher modified H&Y stage were all positively correlated with the not ambulatory status. In addition, domains 1.1, 2.1, 2.3, and 2.5 of FUNDES-Adult showed positive correlation with the severity of walking status (*p* < 0.05).

## 4. Discussion

This is the first study predicting walking status based on FUNDES-Adult. We observed significant statistical differences in age, residence, work status, education level, modified H&Y stage, and FUNDES-Adult scores in each domain among different walking statuses. We also observed higher FUNDES-Adult scores in all domains for more severe walking statuses in patient with PD. Most importantly, residence, modified H&Y stage, and domains 1.1, 2.1, and 2.5 of FUNDES-Adult are independent predictive factors for the not ambulatory status in patients with PD.

Patients with progressive PD experience heterogeneous motor features, having a profound effect on their disability. Increasing age and presentation without tremors may predict rapidly increasing disability; severity of disease and cognitive impairment are associated with motor impairment and future disability [24,25,26,27]. The current study applied FUNDES-Adult to patients with PD and the severity of disability increased as walking status became worse. Though past studies had tried to investigated factors influenced walking ability [28,29], barriers to computation or the comparison of all factors exist for different assessment tools used before. By the end of 2015, WHODAS 2.0 scores had been applied to all known disorders in almost 100 countries cross-culturally [30,31]. The FUNDAS-Adult, derived from the WHODAS 2.0 and ICF code system, is comprehensive, with good reliability and validity. It is a suitable assessment tool for people with disabilities and can be used to measure activities [23].

Our study identified six factors predicting the not ambulatory status in patients with PD. First, being aged >75 years was a positive predictor for the not ambulatory status in patients with PD, similar to the results of a past study [24]. Secondly, we noted that higher modified H&Y stages and scores of FUNDES-Adult Domain 2 were positive predictors for the not ambulatory status. These high scores met our expectation for domain 2, which focuses on patient mobility, and the modified H&Y stage is associated with motor impairment [32]. Third, patients living in an institution may have a higher possibility of being not ambulatory. This may be due to hallucinations and behavioral problems in the patient [33,34], which are more prevalent in patients living in institutions; receiving unnecessary antipsychotic drugs may worsen their motor impairment. Although many factors impact the motor status of patients with PD in an institution, this study is the first to claim that living in an institution predicts the not ambulatory status. Finally, we perceived that the cognition of patients with PD was associated with walking status; the finding is attributable to the progression of disease or compliance to physical therapy.

Physical exercise and increased physical activity improve the performance of activities of daily life and mobility and may reduce mortality in patients with PD [35,36,37]. Exercise improves the functional status of PD. In addition, pharmacological treatment is pivotal to movement disorder, which can improve quality of life and reduce the level of disability in PD [38]. The FUNDES-Adult domains 1.1 (concentrating on doing something for 10 min) and 1.2 (remembering to do important things) represent the cognition of attention and memory function. Our study demonstrated that the severity of impairment of cognitive function can predict walking status; PD with more severe cognitive function may be associated with the not ambulatory status. Postural or gait instability was correlated with cognitive function, and reducing attention and increasing reaction time were both associated with increased fall frequency in PD patients [39,40]. However, memory in this domain did not show similar results, despite the trend in previous data that not ambulatory patients have higher domain 1.2 scores. The prevention of progressing impaired cognitive function may maintain the walking status in patients with PD. However, no studies on preventing not ambulatory status or maintaining walking status by improving cognitive function in patients with PD exist. The current results may aid the development of intervention for mobility in patients with PD to improve walking status.

Patients with PD faced declining mobility as their disease progressed. Our study revealed that decreasing mobility could predict the not ambulatory status using FUNDES-Adult domain 2 scores. We did not analyze domain 2.4 through logistic regression because not ambulatory patients with PD apparently cannot get out of their home. However, we analyzed other items in domain 2, and all items were positive predictive factors for the not ambulatory status. Moreover, home-based training can improve gait and motor function [41,42,43]. Patients moving inside their own home may perform more training at home. Domain 2.3 (moving around inside their home) showed the highest correlation between the FUNDES-Adult score and the not ambulatory status. Based on this result, we assumed that mobility at home could be helpful for patients with PD who are not ambulatory.

This is the first study to use FUNDES-Adult scores for assessing function and disability of patients with PD. However, some limitations exist in our study. First, it is a cross-sectional study, and we could not directly follow-up the ability to maintain walking status of patients with different cognitive functions. Second, the study analyzed disability in patients with PD in Taiwan, and thus, we did not analyze patients with an H&Y stage of <3 because such patients seldom encounter any restriction of daily activity. Third, the less diverse ethnicity of the Taiwanese population and differences in Taiwan’s health care system may lead to differences in walking status.

## 5. Conclusions

The FUNDES-Adult score and residence in an institution were independent predictive factors for the walking status of patients with PD. In addition, it not only is an objective assessment tool for predicting not ambulatory status in patients with PD but also provides reliable functioning and disability summary for these patients.

## Figures and Tables

**Table 1:** Demographic table, *n* = 7455.

Variables	*N*/%	Mean ± SD	*p*-Value ^c^
Gender			0.8439
Male	3719/49.89		
Female	3736/50.11		
Age (years)			<0.0001
18–49	150/2.01	44.20–5.47	
50–64	1434/19.24	59.70 ± 3.84	
65–74	2175/29.18	70.69 ± 2.89	
≧75	3696/49.58	81.19 ± 4.49	
Total	7455	73.25 ± 10.07	
Residence			<0.0001
Community dwelling	6528/87.57		
Institution	927/12.43		
Work Status			<0.0001
Employment	175/2.35		
Unemployment	7280/97.65		
Education level			<0.0001
Above college	240/3.22		
Senior High	850/11.40		
Junior High	1036/13.90		
Primary (include no formal education)	3991/53.53		
Illiterate	1338/17.95		
Family Economic Status			<0.0001
Average	7381/99.01		
middle low & low	74/0.99		
Urbanization level			<0.0001
Urban	3910/52.45		
Suburban	2644/35.47		
Rural	901/12.09		
Domain 8.6 (Motor action)			
Walk for 3 m and return			<0.0001
0	595/7.98		
1	846/11.35		
2	1128/15.13		
3	1325/17.77		
4	3561/47.77		
Modified Hoehn–Yahr Stage			<0.0001
3	2224/29.83		
4	3140/42.12		
5	2091/28.05		
Cognition (domain 1) ^a^			
1-1	7435	2.02 ± 1.42	
1-2	7440	2.21 ± 1.33	
1-3	7321	2.44 ± 1.38	
1-4	6363	2.78 ± 1.28	
1-5	7449	1.50 ± 1.40	
1-6	7441	1.90 ± 1.44	
Mobility (domain 2) ^a^			
2-1	7390	3.23 ± 1.04	
2-2	7453	2.69 ± 1.34	
2-3	7436	2.76 ± 1.33	
2-4	7420	3.18 ± 1.13	
2-5	7267	3.40 ± 0.95	
FUNDES-Adult ^b^			
Cognition (domain 1)	7455	56.76 ± 30.56	
Mobility (domain 2)	7455	80.47 ± 24.80	
Self-care (domain 3)	7455	72.03 ± 28.65	
Getting along (domain4)	7455	65.68 ± 31.09	
Life activities (domain 5-1)	7455	87.57 ± 21.74	
Participation (domain 6)	7455	57.19 ± 24.19	
Summary	7455	67.17 ± 22.02	

^a^ Raw scores; ^b^ Standardized score; ^c^ Chi-square test.

**Table 2:** Demographic table by Walk Status, *n* = 7455.

Variables	Walk Status ^a^			*p*-Value ^f^
	Ambulatory (*n* = 595)	Assisted Ambulatory (*n* = 3299)	Not Ambulatory (*n* = 3561)	
Gender (%)				
Male (*n* = 3719)	8.87	45.07	46.06	<0.0001 ^d^
Female (*n* = 3736)	7.09	43.44	49.46	<0.0001 ^d^
*p* value	0.0077 ^d^	0.3561 ^d^	0.0237 ^d^	
Age (years) (%, Mean ± SD)				
18–49 (*n* = 150)	34.00, 43.44 ± 5.34	54.00, 44.36 ± 5.73	12.00, 45.68 ± 4.47	<0.0001 ^d^
50–64 (*n* = 1434)	19.60, 58.81 ± 4.01	58.86, 59.62 ± 3.86	21.55, 60.70 ± 3.37	<0.0001 ^d^
65–74 (*n* = 2175)	7.77, 69.99 ± 2.86	50.11, 70.62 ± 2.89	42.11, 70.89 ± 2.87	<0.0001 ^d^
≧75 (*n* = 3696)	2.54, 78.89 ± 2.88	34.74, 80.47 ± 4.13	62.72, 81.69 ± 4.65	<0.0001 ^d^
*p* value	<0.0001 ^d^	<0.0001 ^d^	<0.0001 ^d^	
Total	63.84 ± 10.39	71.00 ± 9.98	76.91 ± 8.32	<0.0001 ^e^
Residence (%)				
Community dwelling (*n* = 6528)	8.96	48.38	42.66	<0.0001 ^d^
Institution (*n* = 927)	1.08	15.21	83.71	<0.0001 ^d^
*p* value	<0.0001 ^d^	<0.0001 ^d^	<0.0001 ^d^	
Work Status (%)				
Employment (*n* = 175)	40.00	51.43	8.57	<0.0001 ^d^
Unemployment (*n* = 7280)	7.21	44.08	48.71	<0.0001 ^d^
*p* value	<0.0001 ^d^	<0.0001 ^d^	<0.0001 ^d^	
Education level (%)				
Above college (*n* = 240)	8.33	45.83	45.83	<0.0001 ^d^
Senior High (*n* = 850)	10.71	47.65	41.65	<0.0001 ^d^
Junior High (*n* = 1036)	14.67	52.80	32.53	<0.0001 ^d^
Primary (include no formal education) (*n* = 3991)	7.22	44.88	47.91	<0.0001 ^d^
Illiterate (*n* = 1338)	3.29	33.33	63.38	<0.0001 ^d^
*p* value	<0.0001 ^d^	<0.0001 ^d^	<0.0001 ^d^	
Modified Hoehn-Yahr Stage				
Stage 3 (*n* = 2224)	19.56	61.92	18.53	<0.0001 ^d^
Stage 4 (*n* = 3140)	4.36	49.84	45.80	<0.0001 ^d^
Stage 5 (*n* = 2091)	1.10	17.07	81.83	<0.0001 ^d^
*p* value	<0.0001 ^d^	<0.0001 ^d^	<0.0001 ^d^	
Cognition (domain 1) ^c^ (Mean ± SD)				
1-1 (Concentration)	0.88 ± 1.07	1.50 ± 1.22	2.69 ± 1.31	<0.0001 ^e^
1-2 (Remembering to do important things)	1.19 ± 1.05	1.75 ± 1.14	2.80 ± 1.26	<0.0001 ^e^
1-3 (Problem-solving)	1.23 ± 1.22	1.97 ± 1.26	3.08 ± 1.19	<0.0001 ^e^
1-4 (Learning a new task)	1.55 ± 1.27	2.32 ± 1.23	3.36 ± 0.99	<0.0001 ^e^
1-5 (Understanding)	0.49 ± 0.85	1.00 ± 1.10	2.14 ± 1.43	<0.0001 ^e^
1-6 (Conversation)	0.73 ± 1.04	1.40 ± 1.21	2.56 ± 1.37	<0.0001 ^e^
Mobility (domain 2) ^c^ (Mean ± SD)				
2-1 (Standing for long periods)	1.77 ± 1.28	2.84 ± 1.02	3.84 ± 0.43	<0.0001 ^e^
2-2 (Standing up from sitting)	0.66 ± 0.95	2.03 ± 1.10	3.64 ± 0.70	<0.0001 ^e^
2-3 (Moving around inside home)	0.59 ± 0.85	2.13 ± 1.10	3.71 ± 0.62	<0.0001 ^e^
2-4 (Getting out of)	1.29 ± 1.26	2.80 ± 1.06	3.85 ± 0.44	<0.0001 ^e^
2-5 (Walking a long distance)	1.91 ± 1.28	3.11 ± 0.93	3.92 ± 0.32	<0.0001 ^e^
FUNDES-Adult ^b^ (Mean ± SD)				
Cognition (domain 1)	27.90 ± 23.19	44.90 ± 25.98	72.58 ± 26.85	<0.0001 ^e^
Mobility (domain 2)	37.06 ± 24.94	70.88 ± 21.93	96.61 ± 8.82	<0.0001 ^e^
Self-care (domain 3)	28.97 ± 23.80	59.73 ± 24.78	90.62 ± 16.10	<0.0001 ^e^
Getting along (domain 4)	34.76 ± 28.32	54.77 ± 29.02	80.95 ± 24.63	<0.0001 ^e^
Life activities (domain 5-1)	56.54 ± 29.74	82.07 ± 22.59	97.85 ± 8.63	<0.0001 ^e^
Participation (domain 6)	35.74 ± 19.95	50.60 ± 21.20	66.89 ± 23.05	<0.0001 ^e^
Summary	35.66 ± 18.08	57.84 ± 18.41	81.07 ± 14.37	<0.0001 ^e^

^a^ Walk Status: scores of FUNDES-Adult domain 8.6, 0 = Ambulatory, 1-3 = Assisted ambulatory, 4 = Not ambulatory; ^b^ Standardized scores; ^c^ Raw scores; ^d^ Chi-Square test; ^e^ F-test exact test; ^f^ *p*-value: compared difference of walk status in variables.

**Table 3:** Logistic regression, event = not ambulatory, *n* = 7455 ^a^.

	β	Odds Ratio(Adjusted) ^b^	95% Wald Confidence Limits		*p*-Value
Age (ref. = 18–49)					
50–64	0.357	1.429	0.755	2.705	0.2729
65–74	0.7325	2.08	1.111	3.895	0.0221
≧75	1.2065	3.342	1.791	6.235	0.0001
Residence (ref. = Community dwelling)					
Institution	1.0062	2.735	2.149	3.48	<0.0001
Modified Hoehn-Yahr Stage (ref. = stage 3)					
Stage 4	0.5777	1.782	1.51	2.103	<0.0001
Stage 5	1.5712	4.813	3.946	5.87	<0.0001
FUNDES-Adult Standardized Score					
Cognition	0.00881	1.009	1.006	1.011	<0.0001
Mobility	0.0925	1.097	1.09	1.103	<0.0001

^a^ Stepwise model selection. ^b^ Walk status: Ambulatory or Assisted ambulatory = 0, Not ambulatory = 1.

**Table 4:** Logistic regression, event = not ambulatory, *n* = 6144 ^a^.

	β	Odds Ratio (Adjusted) ^b^	95% Wald Confidence Limits		*p*-Value
Age (ref. = 18–49)					
50–64	0.7268	2.068	0.926	4.62	0.0763
65–74	1.1036	3.015	1.367	6.649	0.0062
≧75	1.5754	4.833	2.2	10.617	<0.0001
Residence (ref. = Community dwelling)					
Institution	0.9376	2.554	1.964	3.321	<0.0001
Modified Hoehn-Yahr Stage (ref. = stage 3)					
Stage 4	0.4128	1.511	1.245	1.835	<0.0001
Stage 5	1.3037	3.683	2.926	4.635	<0.0001
FUNDES-Adult Raw Score					
Cognition Q1	0.2654	1.304	1.203	1.413	<0.0001
Cognition Q2	−0.1878	0.829	0.758	0.906	<0.0001
Mobility Q1	0.258	1.294	1.092	1.534	0.0029
Mobility Q2	0.5845	1.794	1.583	2.033	<0.0001
Mobility Q3	0.7469	2.111	1.833	2.43	<0.0001
Mobility Q5	0.5	1.649	1.342	2.025	<0.0001

^a^ Stepwise model selection, excluded data if independent variable were incomplete; ^b^ Walk status: Ambulatory or Assisted ambulatory = 0, Not ambulatory = 1.
